# Role of General Practitioners in European Health Technology Assessment, EU-HTA Innovation in Oncology as an Example

**DOI:** 10.3390/jmahp14030057

**Published:** 2026-09-21

**Authors:** Daniel Widmer, Hermenegildo Marcos Carrera, Jörg Ruof, Gunasekara Vidanamestrige Chamath Fernando, Patrick Ouvrard, Marie-Christine Bonnamour, Mary McCarthy, Shrikant Atreya

**Affiliations:** 1 European Union of General Practitioners (UEMO), 1000 Brussels, Belgium; 2 Secretariat of the European Access Academy (EAA), 4059 Basel, Switzerland; 3 Institute for Epidemiology, Social Medicine and Health System Research, Hannover Medical School, 30625 Hannover, Germany; 4 Special Interest Group on Cancer and Palliative Care, World Organisation of Family Doctors (WONCA), 1210 Brussels, Belgium; 5 Tata Medical Center, Newtown, Kolkata 700160, West Bengal, India

**Keywords:** health technology assessment, family medicine, public health, drug monitoring, quaternary prevention

## Abstract

Introduction: Different from established clinical guideline workstreams, the participation of general practitioners (GPs) in the health technology assessment (HTA) process at the European level is new. There is a research gap in this domain. The European Union of General Practitioners (UEMO) is the political association of GPs in Europe and has been collaborating as a stakeholder in the creation of the European Regulation on HTA since 2017. The aim of our research is to provide a framework for GPs’role in EU HTA. Methods: Mixed methods: questionnaire and focus groups. Participants: Representatives of national medical/GP associations of 17 Member States of the EU and five EU non-member countries. Results: Most respondents supported GP involvement in the development of clinical recommendations relevant to general practice. Respondents emphasized that UEMO must turn to WONCA-Europe, the academic society of GPs, when scientific input is required. Among the ten priority topics for HTA in oncology, four emerged as most relevant for the GPs: 1. Conscientious prescription of interventions considering over- and under-medicalization, 2. establishing a system for continuity of care integrating palliative care, 3. ensuring evidence-based medicine, and 4. promoting collaborative care and considering workload. Discussion and Conclusions: The GP can contribute to the debate by defending these four priorities. Presenting the difficulties in this debate can contribute to a better understanding of the implementation of new technology in the real world.

## 1. Introduction

Many general practitioners (GPs) consider that the evidence-based medicine (EBM) guidelines do not take sufficiently into account the complexity of their real-world activity and propose a framework to evaluate EBM productions for GPs [1]. This difficult applicability of the guidelines, in practice, encourages the engagement of GPs in their development [2]. The specialist societies that produce recommendations often collaborate in their editorial committees with stakeholders from different groups, including GPs [3,4]. This participation implies new skills and new tools [5]. However, it remains difficult to recruit general practitioners for these panels, the main obstacles being the lack of time and knowledge [6]. GPs’ associations can approve existing guidelines [7] or create them de novo [8]. At the practice level, GPs can discuss the guidelines during continuing education meetings [9] or in local quality circles [10]. They can also benefit from educational materials allowing shared decision-making [11].

**Figure 1:**
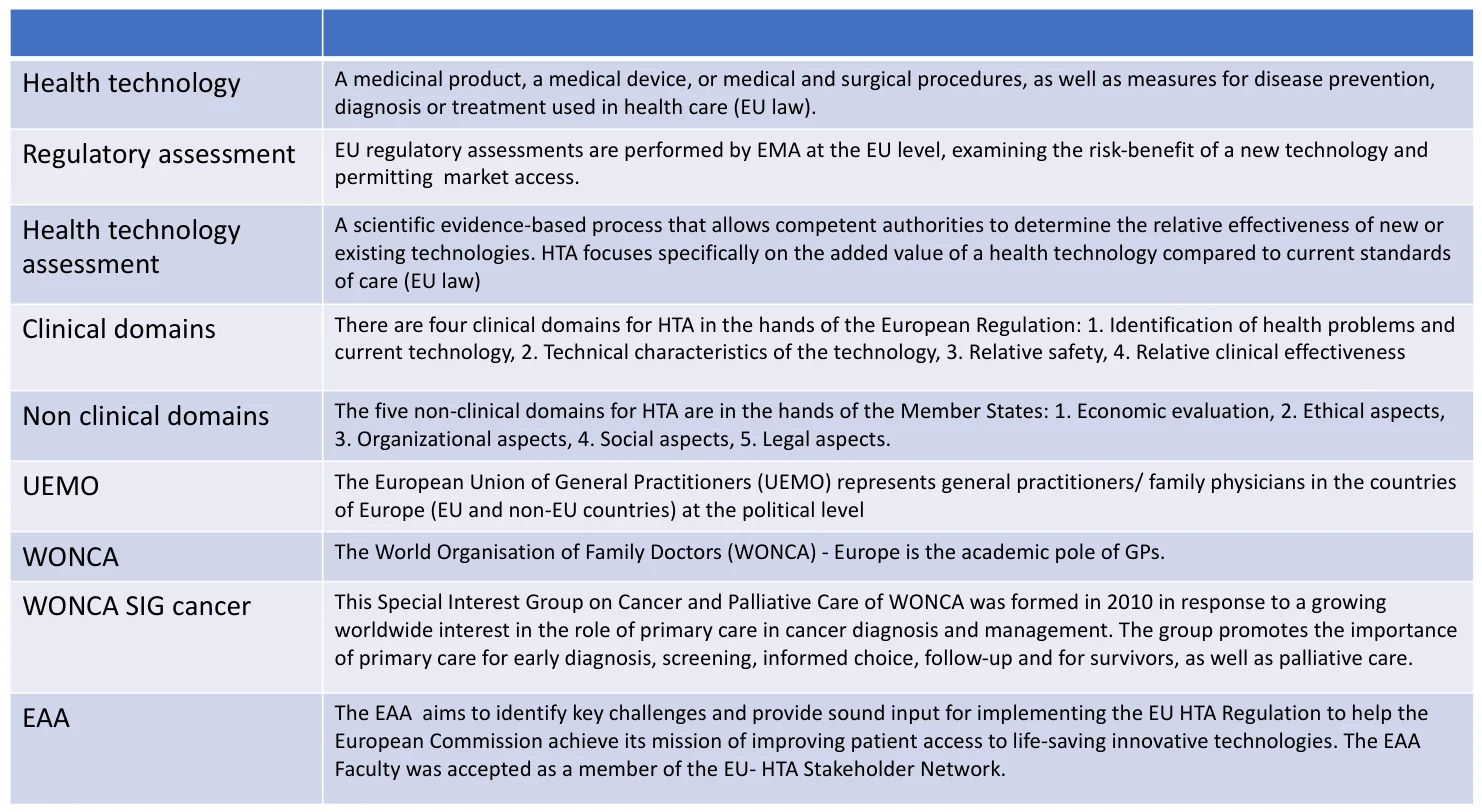
Definition of terms.

If we now look at the development of recommendations at the national and supranational level, we have, on the one hand, the regulatory agencies, which allow market access of new technologies (definition see Figure 1), and on the other hand, the health technology assessment agencies (HTAs), which evaluate the added value. The literature is rarer on the role of general practitioners in this area. I. Heath [12] introduces the situation in 2004. Concerning the UK–NICE system, in 2014 there was a call to consider primary care at all stages of the development of guidelines [13]. NICE defends in principle the voice of primary care [14] in the creation of guidelines, but it seems that in the various national HTA agencies, the use made of the stakeholder’s contribution is not explicit enough [15]. 

The participation of general practitioners at the European level is new. The European Union of General Practitioners (UEMO) [16] represents general practitioners (GPs)/family physicians in the countries of Europe (EU and non-EU countries) at the political level. Since 2017, UEMO has been a stakeholder in developing the European Regulation on health technology assessment (HTA) [17]. Since December 2021, UEMO has been an official member of the European Commission (EC)’s EU-HTA Stakeholder Network and has been supporting the implementation steps. UEMO has also collaborated closely with the European Medicines Agency (EMA) [18] since 2019. During internal discussions, UEMO provided four key reasons for its participation in the EU-HTA process: 1. GPs are important prescribers (50% or more, according to countries). 2. New technologies can redefine the role of GPs in the future. 3. Political regulation is necessary, and GPs must participate in the construction of this regulation to support the translation of evaluated (internal validity) innovative and technical capabilities into the reality of patient care (external validity). 4. The European regulation is necessary to foster collaboration and harmonize HTA across Europe. With its development, the EU level will become more important in setting the standards.

Since January 2025, the first assessments have started, with Oncology and Advanced Therapy Medicinal Products (ATMPs) being the initial focus of EU-HTA. From 2028 onwards, any orphan medicines will have to undergo EU-HTA assessment; a full scope assessment is planned from 2030 on. Twelve Joint Clinical Assessments (JCAs) have started in 2025, with ten thereof addressing oncological conditions. Until 2028, most EU-HTA assessments will cover oncology medicines, making oncology the «pacemaker» for the evolving EU-HTA process. The assessment needs experts for input on topics requiring a high level of respective specification. Various stakeholders (patient’s, clinician’s, payer’s and industry’s associations) are involved in EU-HTA; however, stakeholders are not experts but can defend general principles and provide advice. With relevance to family medicine, the question is whether there is a place for GP experts to defend comprehensiveness and continuity of care. During internal UEMO debates, various topics were raised about the opportunity and modalities for continuing the process of collaboration, with the political role of stakeholders in monitoring the implementation of the law, or even accepting the role of an expert in the evaluations, considering that UEMO is not a scientific or academic organization. This study collects the opinions of the UEMO members with pro and contra arguments. In preparing this report, it was necessary to enlarge the vision with two other associations, the WONCA [19] Special Interest Group (SIG) on Cancer and Palliative Care [20] for the scientific aspects and the European Access Academy (EAA) [21] for a pluri-stakeholder vision.

For the role of GPs in cancer, we can rely on the principles defined by the SIG (Figure 1) and on data from the literature showing the importance of the general practitioner throughout the curative treatment of cancer from the point of view of the patient [22] and the GPs themselves [23], for the coordination of care [24], for consideration of the trajectory of the disease [25], and for psychological support [26], including that of the family and caregivers [27], which continues after the death of the patient, maintaining a holistic approach.

According to the European Regulation (17), evidence-based medicine (EBM) is the backbone of the EU-HTA process. Within EBM, the healthcare provider’s clinical perspective is one of the three constituting components, beyond the patient’s perspective and research evidence. As GPs are the only clinical experts involved across all medical conditions, their consolidated input is decisive to invigorate the clinical perspective and to ensure a balanced implementation of the EBM triad within the evolving EU-HTA process. Therefore, the objective of our research was to identify basic principles of GPs’ involvement in the EU-HTA process and to prioritize them. What can be the role of GPs in EU-HTA?

## 2. Methods

**Figure 2:**
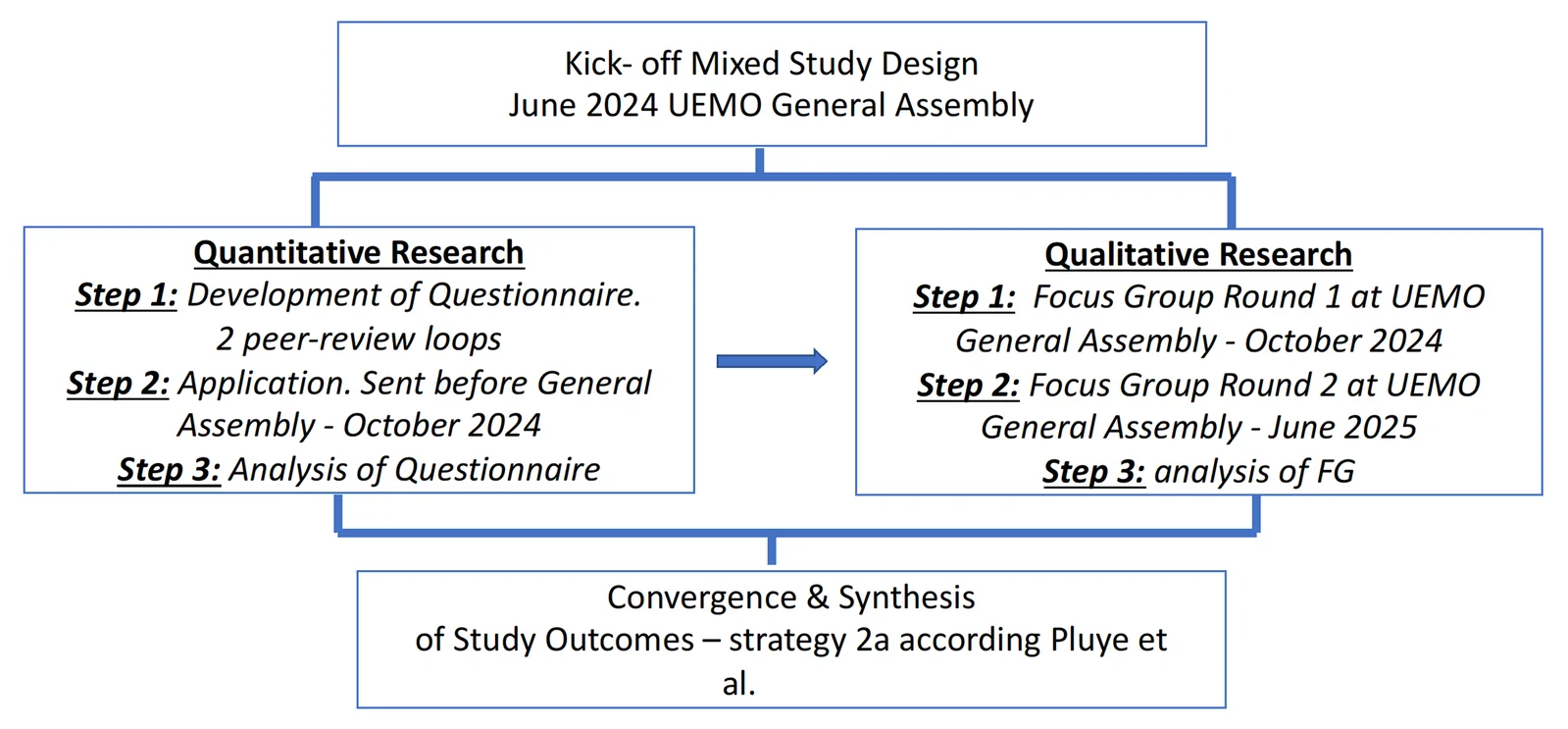
Mixed study design combining qualitative and quantitative elements [28,29].

An overview of the study design is displayed in Figure 2. The study, aiming to determine the role of GPs in HTA in the field of oncology, was conceived after seeking input from UEMO and discussed in June 2024 at the UEMO General Assembly in Stavanger (see Supplementary Material research protocol). A mixed method was applied, using a convergent design [28,29,30]. (See Supplementary Material research checklist COREQ and MMA). The mixed methods consisted of a quantitative and a qualitative research arm that were finally converged to reveal the final outcomes.

**Quantitative Research:**

The collection of quantitative data relied on a questionnaire-based approach, including a limited number of ordinally ranked Likert scale items. A draft questionnaire was developed by D.W., H.M.C., M.M.C. and P.O. and passed through two iterative peer-review loops before finalization: (i) virtual peer review by discussing with UEMO (D.W., H.M.C., M.M.C., P.O.), WONCA-SIG cancer (A.S., C.F. and 2 other members) and EAA (J.R.) experts by Zoom meetings (Zoom Video Communications, Inc., Version 6.6.9) and email correspondence (all authors), and (ii) discussion of advanced draft during the WONCA congress in September 2024 [31,32] in Dublin. Subsequently, the finalized questionnaire was presented to 27 UEMO members and observers during the General Assembly (GA) on 17 October 2024 and on the internal website. For the questionnaire, one response was collected per country.

Four UEMO members were not present at the GA and were therefore unable to complete the questionnaire, but were informed via the internal website. One member was present and did not respond. Only descriptive analytics (frequencies and percentages) were applied. According to the usual rules of UEMO, it is not possible to make an analytic analysis per country since, during the discussions, representing members express themselves and not as the official position of the national medical organization. Analyses were conducted by DW and HMC.

**Qualitative Research:**

The conduct of focus group (FG) discussions is a standardized procedure throughout the UEMO conventions. As per the usual methods of work at UEMO, the FGs were conducted on the first day of the General Assembly and allowed the investigators to obtain the perspective of the members. The first FG of 16 participants occurred at the UEMO GA (16 October 2024), moderated by D.W. and with H.M.C. as an observer, both experienced in qualitative research. The sampling method for the FG was based on convenience, i.e., members attended because of their interest in the topic. Focus groups were audio recorded (duration 90 min) and transcribed verbatim. The focus of the discussions was based on the questionnaire (the questionnaire was presented for introduction at the first FG without knowing the answers of the members). The coding process of the FGs (thematic analysis—themes identified from the data) [33] was conducted with MAXQDA 2018 and two coders (D.W. and H.M.C.) (See Supplementary Material list of codes Document MAXQDA 2018). A second focus group with 13 participants was organized at the beginning of GA in Helsinki, June 2025. The coding process of this second FG (D.W. and H.M.C.) found the same codes as in the first FG and seven new codes in the thematic analysis, for a total of 45. It is always difficult to assert that the saturation of data was obtained: we can say that the second FG took place before the GA in Helsinki, and the results of the questionnaire and the first FG, plus the global results of the second FG before coding, were presented to all members without any more comments. We note that 10 countries out of 22 did not participate in the two FGs.

In the FG, two or more representatives per country could join. Most participants were actively practicing as a GP in their country. UEMO delegates are generally mandated by their respective national member association. Quotes from FG discussions are referred to by a number in brackets attributed to each participant as GP1, GP2, etc. Four UEMO members were not present at the GA and were therefore unable to participate in the FG, but were informed via the internal website (no answer from those members). One member was present and did not respond to the questionnaire; however, this member expressed himself in the FG.

Throughout the analysis of the FG outcomes, responses were clustered and grouped into those that are supportive versus not supportive of having GPs involved in HTA-related discussions.

**Convergence and Synthesis of Outcomes.**

Both qualitative and quantitative research outcomes were reviewed by all co-authors. Results were discussed and clustered with the help of a comparative table (strategy 2 a. according to Pluye [28]: comparison of results obtained separately) in a final set of priority domains that GPs should be representing when engaging in HTA-related discussions.

## 3. Results

We will first present the construction of the questionnaire, then the results of the mixed methods, with a convergent design, by analyzing the questionnaire and the FGs separately, and then synthesizing the two.

**Construction of the questionnaire:**

We considered the different levels of the recommendations for clinical general practice, from the less visible to the more visible (Table 1): European regulatory body (EMA) and EU-HTA, National regulatory bodies and HTA, guidelines from GPs’ societies and from specialist societies, continuing medical education, and elaboration of recommendations in quality circles, and for shared decisions.

We also discussed the crucial topics for GPs in the evaluation of new technologies in cancer (Table 2): to be attentive to quaternary prevention (the principle of non-maleficence, first do no harm) and over-medicalization; to consider conflicts of interests for nomination of assessing experts; to consider the workforce necessary for technology implementation; to consider the need for continuity of care, to take into account the long-term side effects for survivors; to apply EBM principles; to edit guidelines in plain language and permit shared decisions with the patient; to consider inequity of access to technologies in remote or deprived areas; to consider palliative care as an alternative to be discussed or as a comparator; to promote collaboration between GPs, oncologists, patient, team, family and carers; and to consider that PICO (population, intervention, comparison, outcomes) can be different in general practice with a specific population, interventions that are not always technological but can use communication or relationship, non-technological interventions such in palliative care that can be the comparator, and outcomes with an accent on quality of life.

**Analysis of questionnaire:**

The responses to the questionnaire (Table 3) indicate that the majority of UEMO members would prefer GPs to participate in the development of clinical recommendations (85% quoting 5), with an interest in specific GP societies’ guidelines (86% quoting 5 in Table 1), national regulatory bodies (77%) and national HTA (64%). There is more ambivalence at the European level (Table 1). The vision GPs want to introduce, in the focus group debates expressed as ethical dimensions, holistic dimensions and workload, is probably rather considered at national-level HTA than at EU-HTA, which is centered only on clinical dimensions (definition, Figure 1). Involvement of GPs in assessments is clearly encouraged by UEMO members (73% quoting 5 in Table 3), with a trend to collaborate with WONCA and WONCA-SIG for identifying experts. Four items are prioritized in the identified points to address in the evaluations of new cancer technologies (Table 2): the risk of over-medicalization (77% quoting 5), the importance of continuity of care and attention to long term side effects (77%), the application of EBM principles (77%), and good collaboration within the team (68%).

**Analysis of FG:**

Table 4 shows the arguments in favor of the participation of GPs in HTA, for those who think it is essential to be part of a process that is “going to happen anyway” (GP5). We must introduce our vision to the politicians who “sometimes just don’t know” (GP19), to provide our practical advice (GP14) within the scope of our practice (GP1). For example, as in our consultations, we frequently see patients with rare diseases (GP2), and rare diseases are within the scope of our practice. GPs are also at the forefront of observing new epidemics: a participant of the FG provides the example of the latest treatments for obesity where GPs “should have training and accreditation to prescribe and be actively involved” (GP7). The presence of GPs in the discussion is also vital for an ethical dimension (GP3), a holistic vision (GP14), for defending the quality of life (GP3), for presenting the workload generated by new technologies and “how to overcome this” (GP4). In oncology, the continuity of care and the risk of over-medicalization are emphasized, as in the questionnaire, even if “over-medicalization is always linked to under-medicalization, because the resources are limited in most of our countries”. (GP19). With the defense of EBM, GPs can also have a role in the orientation of research (GP3) and can defend the vision of the patient (GP18). If the GP has an active role in elaborating clinical recommendations, their application could be better (GP1). Finally, if it seems important to identify GP experts for evaluation with the help of the academic pole WONCA (GP8), the role of UEMO stakeholders is also important for bringing “a voice of sense to experts” (GP15).

Table 5 provides the results of the FG who expressed fears and arguments against the participation of GPs in the HTA process. As the process is far from daily practice (GP4) and seems to be very complex for GPs who consider themselves experts in “commonalities” (GP4) and in communication before technology (GP2), it was essential to have this open discussion. Further, the topic is considered “a niche” and perhaps only “the more academically driven ones” (GP11) with special interests (GP5) can be involved. There follows a list of problems related to the HTA process itself, which are not included in the competencies of GPs: conflicts of interest (GP5), economic questions (GP4), industrial confidentiality (GP5), reductionist vision of the PICO (GP3), and experts used to “rule out political dimensions” (GP19). “What is the cost of a collaboration for GPs? Can basic GPs do this job in terms of training and funding? Who should pay the GP stakeholder or expert?” (GP14). “And what can we expect in return?” (GP5). The fear of being instrumentalized as an alibi was also expressed (GP22). For the question of oncology, to have a discourse on palliative care seems more critical to GPs than drugs and devices, because our role is to be “engaged with our patients to the end” (GP12) and beyond for the family (GP14).

**Common synthesis:**

Table 6 combines both quantitative and qualitative approaches based on the four main points identified by the questionnaire: over-medicalization, continuity of care, EBM, and collaboration. We see the creation of a new interpretation considering the qualitative insight.

**Needing conscientious prescription of interventions considering over- and under-medicalization.** While the questionnaire highlights the risk of over-medicalization, the discussions also address under-medicalization, the ethical dimension, and consideration of quality of life in the evaluations. Additionally, fears about the complexity and burden of the process are addressed: “This is much above the level of what an average GP will be able to do” (GP4). This conscientious position requires honesty and modesty: we must defend “real life” before the “scientific position” (GP3).

**Establishing system for continuity of care integrating palliative care**, from the diagnosis to palliative care and beyond (GP12). “GPs, after the treatment, will be there for the patient and have side effects to manage” (GP2). No continuity without communication before technology (GP2). Communication described by a participant as “the black hole of the profession” (GP10).

**Ensuring a balanced implementation of the three fundamental principles of EBM and avoiding a reductionist vision:** GPs emphasize professional experience, which is one of the three dimensions of EBM alongside science and patient experience. “It is extremely important to have working doctors from primary care to provide practical advice for the proper implementation of HTA. Also, for asking questions” (GP14). GPs “bring a voice of sense to the experts, that they are the common-sense doctors” (GP15).

**Promoting collaborative care considering workloads for all professionals and carers:** From the screening to the end: “We are not experts in oncology. But we can give input to say that it is important to think that GPs… will be there for the patient… to the end of the process.” (GP2). “Collaboration between the oncologist and GP team, nonprofit…organizations… and family produces the best results together with spiritual or religious support (GP14). 

We were not surprised to find these four priorities at the top of our results, as they are regularly discussed at UEMO and WONCA. More surprising is the seventh-place ranking for equity, perhaps because all respondents are European and the issue of access to new technologies for everybody is less of a concern for them within the framework of legislation that addresses this question. Attention to under-medicalization appears in the verbatim for patients who could not fairly access the necessary treatments. In the preliminary discussions for this study, general practitioners were reluctant to engage in such a complex field, and we were surprised by the willingness to become involved expressed by the quantitative results. The verbatim comments tempered this enthusiasm, revealing a fear of a process that could impact the already heavy workload of general practitioners. We can speak here of a paradox: there is a wish to defend the practice of basic GPs, but there is no question of entering a niche, even an academic one, for fear of being out of touch with reality. This constitutive paradox of general medicine will be discussed later.

## 4. Discussion

### 4.1. Necessity to Participate in EU-HTA

As pointed out in the introduction, integration of the healthcare provider’s clinical perspective is critical to a successful implementation of the EU-HTA Regulation (EU HTAR). Any HTA-related effort is based on the principles of EBM, and within EBM the clinical perspective, i.e., GPs’ and specialists’ input, is one of the three constitutional elements [17]. However, besides providing their clinical perspective into the assessments, GPs are usually the central point of care where all three EBM components (research data and evidence; clinical perspective and experience; and patient perspective) are integrated. This is another good reason for GPs to participate in EU-HTA.

Of note, already in the construction phase of the legislation, the discreet position or even the withdrawal of clinicians and payers was noted in favor of pharmaceutical companies and patient associations during the discussions [34]. It is therefore important that clinicians make their voices heard alongside patients [15,35], so that conclusions are not based solely on paper knowledge [36], with the risk that the presence of stakeholders becomes an alibi, as some of our participants fear. The commitment of all stakeholders is faced with constraints regarding capacities and expertise on the subject [37], as our participants noted. In Canada, observers also mentioned the lack of knowledge on HTA processes among practitioners and the difficulty for GPs to acquire skills in a field apparently far removed from practical work [38]. What can we expect in return? HTA work must also lead to concrete consequences for practice [39,40] in the form of useful guidelines.

### 4.2. What Is the Role of GPs at EU-HTA? (Figure 3 Conceptual Framework)

First, we must distinguish the stakeholder role from the expert role. This study presents the opinions of the UEMO, who has a political stakeholder role in the elaboration and implementation of the Regulation. Discussions are underway with WONCA to find out if the academic association could engage in the role of a scientific expert for the evaluations. UEMO certainly has an important role of advocacy for GPs. Alongside the presentation of the priorities of general medicine to contribute to the construction of guidelines, the advocacy highlights certain difficulties important for the feasibility, such as professional overload with the risk of burn-out, lack of resources, and responsibilities required by new technologies, as well as their final impact. Even if the European Regulation only considers the clinical aspect, we must anticipate these problems at the European level for future guidelines at all levels. This is the whole challenge of articulating EUHTA with real practice.

**Figure 3:**
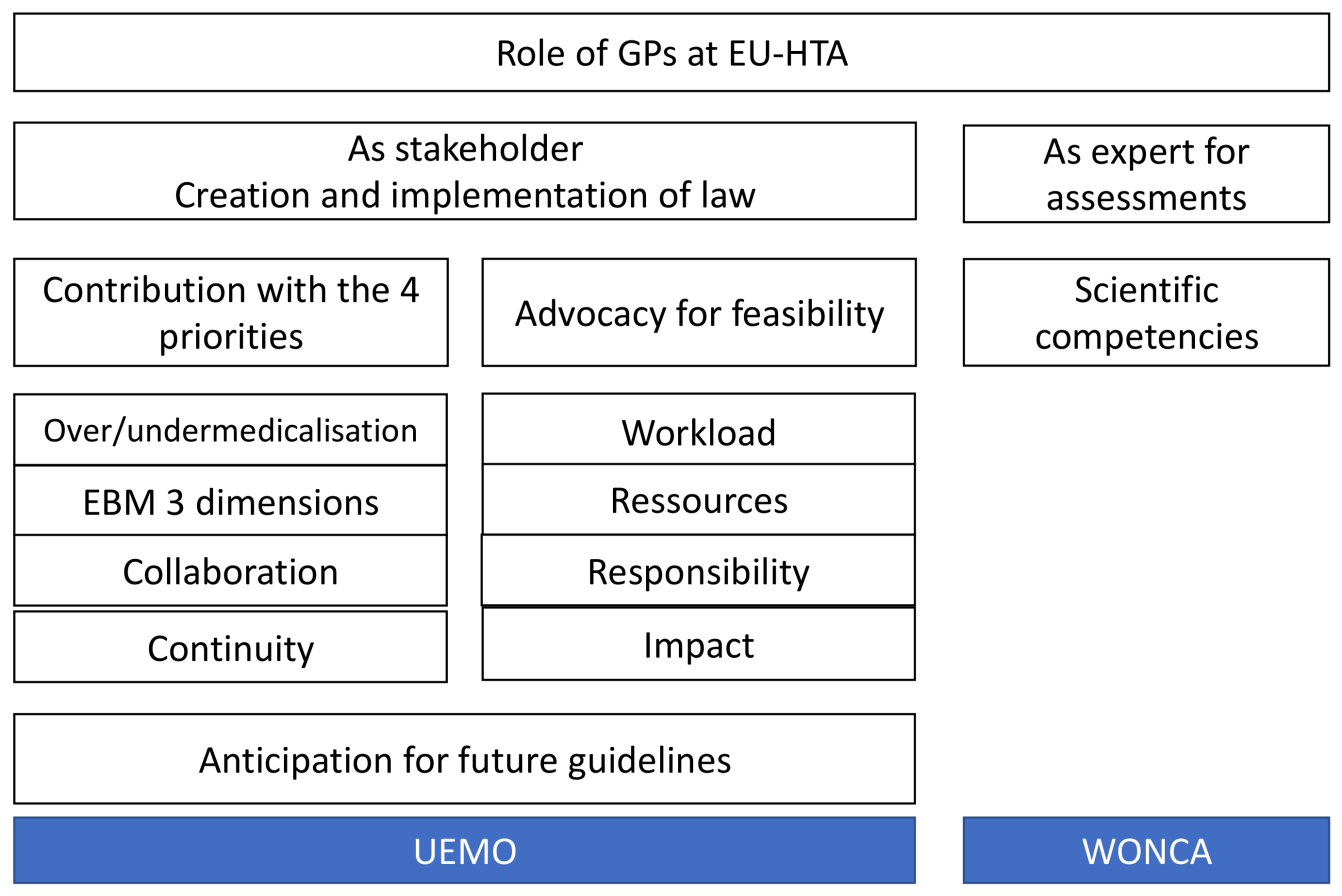
Conceptual framework.

Twenty years ago, our colleague Iona Heath [12] feared that HTA was delivering less than what it promised. It is worth reviewing her comments after the legislative process in which we participated as GP stakeholders. Like her, we can welcome a European system, which avoids the duplication of effort between different European countries. As she suggested, we were able to see from our place as UEMO in the debates the different views of stakeholders and the extent to which “deliberation should not disappear in front of a facade of rationality”. General practitioners are first confronted with individual needs rather than with the “utilitarian agenda of public health,” and it is understandable that some might think that HTA is not a priority for GPs. This is perhaps particularly true at the European level, which delegates the social and ethical dimensions to Member States. These dimensions, as Heath states, “cannot be marginalized in general medicine in favor of measurable linear relationships between input and output”. However, if we do not want a super-rational form of HTA to “exercise inadequate control over professional behavior”, we must be present as GP stakeholders in the debates and defend HTA decisions that are useful for the practice of GPs [41] as tools for shared decision-making, such as the number needed to treat (NNT) and the number needed to harm (NNH) [12]. Iona Heath further requests that we also consider multimorbidity and vulnerable groups and the risks of prevention technologies, which today take the form of genetics and so-called personalized predictive medicine. Quaternary prevention is a core concept of family medicine and was defined by WONCA in 1985 as an “Action taken to identify patient at risk of over-medicalization, to protect him from new medical invasion, and to suggest to him interventions, which are ethically acceptable”. In the context of HTA, it could be possible to falsely assign an additional benefit (type 1 error corresponding to over-medicalization) versus not recognizing an existing additional benefit (type 2 error corresponding to under-medicalization). With some shifting of evidence paradigms (introduction of single-arm trials in oncology), the balance between error type 1 and 2 could be more difficult to maintain [42].

As we can see in our conceptual framework, there is a gap in the definition of scientific competencies for future experts’ general practitioners, since this was not the focus of our research, which centered on general themes that we prioritized as stakeholders. The question is how to articulate these themes with the skills of the future expert. These experts will need to become familiar with HTA methods, such as Regulatory Impact Assessments RIA [43,44] or Environmental Impact Assessments EIA [45,46], which stem from a public health perspective. Concerns can converge between public health and general practice, particularly when it comes to combining clinical and non-clinical dimensions [47], as well as addressing equity [48,49] and the environmental impact of new technologies [50,51]. However, the values are not entirely the same between the distributive justice of public health and patient-centered medicine [12], which practices a justice of recognition [52]. The key question will be how to translate HTA findings into clinical practice. Clinical epidemiology is important in this regard, and practitioners will request NNTs (Number Needed to Treat) rather than relative risks [12]. A translation movement in the hands of clinicians must develop, equipped with practical tools [53] and a better understanding of environmental issues in the office [54]. This translation will require knowledge [55] and new methodologies; the role of data from general practitioners in the RWE (Real World Evidence) is also being considered [56,57]. Some authors have spoken of epistemic injustice when the bottom-up view of practice is left in the shadow of mainstream science [58,59,60]. A translation of the public health vision into terms compatible with practice makes it possible to confront the paradox that we have described and will enable the engagement of general practitioners in the HTA process.

### 4.3. Strengths and Limitations of the Study

The strength of the study is that it was able to collect the opinions of doctors from many European countries who, in addition to their political activity at UEMO, work as general practitioners.

A limitation of this study relates to the sample of participants included. All of them are UEMO delegates and/or national representatives, i.e., are involved in ongoing scientific and political discussions. Such proximity may introduce a confirmation bias that has to be considered when reflecting on the study outcomes. We took care to minimize this throughout the research process through triangular discussions with the co-authors, two of whom are members of other European organizations (EMA for MMC, HERA for PO and EAA for JR), one is a lawyer (MCB), and two are members of a scientific organization (WONCA for AS and CF). Furthermore, the research process was discussed in UEMO workshops and General Assemblies, where opinions differed regarding general practitioners’ engagement within HTA. The questionnaire was discussed at a WONCA workshop in Dublin with European general practitioners not involved in HTA. The main confirmation bias stems from the defense of the profession, given that all the authors except MCB and almost all participants in the FG are general practitioners convinced of the usefulness of their profession. Advocacy is certainly an essential dimension of the practitioner’s activity. When GPs conduct research without the participation of patients or other professions such as sociologists or anthropologists, they must be aware of the importance of their tendency to advocate.

Therefore, the next steps on the research agenda are (i) further shaping and advancing the scientific and political contribution of GPs to EU-HTA and (ii) integrating such contributions in the wider professional and stakeholder network to further strengthen GPs’ unique and central role in healthcare, where all elements of EBM, i.e., patient-centric care, scientific evidence and clinician’s perspective and experience are integrated [61].

## 5. Conclusions

After this open debate facilitated by the questionnaire and the two focus groups, the UEMO General Assembly was able to vote unanimously for the continuation of the collaboration with EU-HTA as a stakeholder and for asking WONCA if there are future academic resources to find experts for evaluations.

We hope that this study will be useful for general practitioners by introducing them to a little-known area and to the possible articulation of different levels in the development of clinical recommendations used in daily practice. We also hope that they will be able to translate the advocacy of the profession into data that speak to public health managers, as WONCA has already done with the European atlas of general medicine [62]. We hope that this paper will generate greater commitment from general practitioners in Europe, both regarding stakeholders and future experts for evaluations through the academic help of WONCA. Public health and HTA managers should also understand that if GPs are committed to collaborating in the HTA process, they will always be concerned with the ultimate consequences for their practice and will anticipate the non-clinical, social, and ethical dimensions of HTA conclusions. Let us hope that, through their professional advocacy, GPs inform managers about the feasibility of new technologies, particularly in terms of workforce.

New technologies and the consideration of genetics, for example, will also shape the general practitioner’s work. When a patient undergoes genetic testing related to her/his cancer, what will the GP do with the family members who consult her/him? She/he will need education to answer questions and to provide useful advice on prevention and screening. She/he will also be responsible for the medical situation of survivors and for palliative care. It is therefore important to seek out GP experts whenever a new technology is likely to change primary care practices. The exercise carried out with oncology has allowed the GP community to prepare for the future of HTA.

There is also place for future research questions: relationship of guidelines and HTA? Contribution of GP perspective into HTA? Implementing HTA outcomes into GP clinical practice? Guidelines for cross-functional collaboration to improve continuity of care. The future of GPs’ research in this domain will depend on the academic interest of the institutes of general practice. There will also be room for interprofessional research between health care professionals and interdisciplinary collaboration to better contextualize advocacy. Let us not forget that psychiatrists [63], philosophers [64], and anthropologists [65] have highlighted the “apostolic function” of the doctor.

## Figures and Tables

**Table 1:** Quantitative Research. Outcomes of the Questionnaire Part 1 addressing areas that GPs should be involved with. If you consider that participation of GPs is necessary to the elaboration of recommendations, how do you assess its importance at each level? From 1. Unnecessary to 5. Essential. 27 questionnaires distributed: 22 countries answering–5 non answering.

	1	2	3	4	5	No Resp. to the Question	% Nbr 5/22	Priorities
Documents for shared decision	2		4	4	11	1	50	6
Quality circles and Continuing Medical Education		1	5	2	14		64	3
GPs societies guidelines			2	1	19		86	1
Specialist societies guidelines			6	6	10		45	7
National HTA			1	7	14		64	3
National regulators bodies		1		2	17	2	77	2
EU-HTA	1		4	4	12	1	54	5
EMA	1		1	7	13		59	4

**Table 2:** Quantitative research. Outcomes of the questionnaire Part 2 addressing what GPs should be attentive to when faced with new technology for cancer? From 1 not important to 5 very important-? Don’t know. 27 questionnaires distributed: 22 countries answering–5 non answering

	1	2	3	4	5	?	No Answ.	% Nbr. 5/22	Priority
Overmedicalisation–first do not harm	1	2		1	17	1		77	1
Conflicts of interest for the nomination of assessing experts	3	1	5	5	5	3		23	8
Workforce necessary for technology implementation	1		5	4	8	4		36	7
Continuity of care/long term side effects/survivors	1				17	3	1	77	1
EBM principles	1		1		17	2	1	77	1
Guidelines expressed in an understandable language for patients, permitting shared decisions	1	1		3	13	3	1	59	3
Consideration of inequity of access to technologies in remote or deprived areas		1	2	5	9	5		41	6
Early palliative vision: consider palliative care as an alternative to be discussed	2	1	1	2	12	4		55	4
Collaboration GP-oncologist-patient-team-family-carers	1		2		15	3	1	68	2
Attention to PICO in assessments–choice of comparator and outcomes	2		5	1	10	4		45	5

**Table 3:** Quantitative research. Outcomes of the questionnaire Part 3 addressing who is best for scientific evaluation of new technologies at EU-HTA level. From 1 not agree to 5 totally agree. 27 questionnaires distributed: 22 countries answering – 5 non answering

	1	2	3	4	5	No Answ.	% Nbr 5/22	Priority
GP must participate to the development of clinical recommendations that they should use in their practice				3	19		86	-
UEMO must encourage the presence of GPs in the assessment process of new technologies				6	16		73	-
Who is the best: WONCA Europe	1	1	1	3	14	2	64	2
Who is the best: WONCA Special interest group		1		5	15	1	68	1
Who is the best: Specialist societies	2	1	4	5	9	1	41	4
Who is the best: University Departments of GP	1	3		4	13	1	59	3
Who is the best: Pharma industries	9	3	1	1		8	-	-

**Table 4:** Focus group analysis. Arguments in favor of participation of GPs in HTA.

Codes	Sub-Codes	Verbatim
To be in the process		I would fear not to be part of it. (GP5)
Inform the political level		… to place our opinion and influence at that level…. but sometimes it’s that they just don’t know. (GP19)
	For practical advice	… to give practical advice. For the proper implementation of HTA. Also, for asking questions. (GP14)
	For scope of our practice	Our advice is, is within our scope of practice. (GP1)
	On ethical dimension	On ethical points, I guess that we have a role in ethics Committee (GP3)
	For rare diseases	One patient on 18 has a rare disease in our consultations, … (GP2)
	For discussing everyday practice	The new epidemics, e.g., obesity …. the iceberg of disease, … and we should have perhaps training and accreditation then to prescribe and be actively involved. (GP7)
	For quality of life	… we’re going to ask about quality of life of patient in this kind of situation. (GP3)
	For holistic dimensions	The experience of GPs is holistic and is essential in development of recommendations which will determine their mode of practice (GP14) I find that the collaboration between oncologist-GP team (navigator nurse), … voluntary organizations and family, produce the best results together with spiritual or religious support. (GP14)
	For better application of guidelines	… not many GPS get involved with the NICE guidelines, and which is why GPS tend to interpret many of them that apply to general practice issues with caution, because they’ve not properly been consulted or involved. (GP1)
	For discussing workload	important for GPs to highlight barriers to implementation due to extra overload and suggest how to overcome this. (GP14)
	For orientation of research	We get the green light to say, okay, you can go and get your research, or the red light and say no. (GP3)
	For continuity of care	We are not experts in oncology. But we can give input to say that it is important to think that GP, after the treatment, will be there for the patient. To give such input, even at the European level. (GP2)
	For considering over and under-medicalization	Over-medicalization always is linked also to under-medicalization, because the resources are limited in most of our countries (GP19)
	For defending position of patients	How is the patients experience? E.G., the Proms (patient-reported outcomes measures) and the Prems (patient-reported experience measures). (GP18)
The importance of being stakeholder		The EC has considered important to invite stakeholders to be consulted… so it does not mean that the stakeholder will carry out all the HTA work (GP6)
WONCA for expert/UEMO for stakeholder		Also, WONCA, it is really the organ. (GP8) I think the value of UEMO … to be engaged with HTA is that they bring a voice of sense to the experts, They are the common-sense doctors… (GP15)

4 persons participated to both FG. FG 1: with 16 persons from: Portugal–Serbia–Finland–France–Italy (2 persons)–Ireland (2 persons)–UK (2 persons)–Cech Republic–North Macedonia–Slovenia–Spain–Switzerland and UEMO jurist. FG 2: with 13 persons from: Spain–Cech Republic–EMSA (students in medicine)–Finland (2 persons)–Italy–Switzerland (2 persons)–France–UK–Portugal–one person non readable (no speech)-UEMO jurist.

**Table 5:** Focus group analysis. Fears and arguments against participation of GPs in HTA.

Codes	Sub-Codes	Verbatim
Fear to be taken as an alibi		How much real change are we affecting? (GP4) Better not to participate than to be taken advantage of. (GP5) What it means in regard to tokenism (GP22)
Fear to be engaged in a too complex process	With risk of conflict of interests	We must be aware that this is a big money behind, and the conflict of interests may be great. (GP5)
	With questions about the representation of patients	The best person representing the patient is that patient or a patient’s body? (GP4)
	With economic questions	It’s in Department of National Pharma Economics who do these health technology analyses. (GP4)
	With problems of industrial confidentiality	I was not allowed to talk about it with anybody else because it would be confidential, and cause was big money. (GP5)
	With too much responsibilities	And then, are you insured for that? If You become responsible? (GP4)
	With a too reductionist vision	PICO is just a tool to build a good research (GP3).
	With too important top experts	The government … take some top experts: but you have no idea of the health system, of the health politics, and so on. And then you put down some rules, and then we have, afterwards, the problems… (GP19)
Fear of the cost of the process	In terms of resources	Above the level of what an average GP will be able to do (GP4) It is perhaps a topic who is too complicated even for an institute of general practice. (GP2)
	In terms of funding	GPs should participate in developing guidelines relating to day-to-day practice and be … remunerated to this. (GP14)
	In terms of return	We should say what we are expecting in return of collaborating to… (GP5)
Fear that a niche interest makes us forget priorities:		I think this will always be a niche kind of field… it will entice some GPs interested, …. the more academic ones. (GP11) GIPSI, GP with special interests… who would get some extra education and extra knowledge. (GP5)
	Daily practice	And so, I’m not too sure where this fits into me in my daily general practice, other than to know it exists. (GP4)
	Commonalities	We’re experts in commonality. Commons conditions. (GP4)
	Communication	Another thing is the communication… it’s very a black hole for the profession (GP10) Speak with the patient before technology? (GP2)
	Palliative care	I am also involved in palliative care because we are engaged with our patients to the end…. Meanwhile, with drugs or some devices, I don’t think that it is our role. (GP12)

**Table 6:** Convergence and synthesis of qualitative and quantitative study outcomes: four priority domains that GPs should be representing when engaging in HTA-related discussions.

Priority Domains	QUANT Results	QUAL Results—See Codes	Interpretation
Overmedicalisation—first do not harm	17/22-77%	On ethical dimension For quality of life Considering workload Over and under-med.	Needing conscientious prescription of interventions considering over **and under-medicalisation**
Continuity of care/long term side effects/survivors	17/22-77%	Continuity of care Communication Palliative care	Establishing system for continuity of care **integrating palliative care**
EBM principles	17/22-77%	Introduce our vision Practical advice Scope of our practice Better applic.of guidelines Orientation of research Cave reductionist vision	Ensuring a balanced implementation of the three fundamental principles of EBM and **avoiding reductionist vision**
Collaboration GP-oncologist-patient-team-family-carers	15/22-68%	Holistic dimensions Considering workload Defending patients	Promoting collaborative care **considering workload** for all professionals and carers

## Data Availability

The raw data supporting the conclusions of this article will be made available by the authors upon request.

## Supplementary Materials

The following supporting information can be downloaded at: https://www.mdpi.com/article/10.3390/jmahp14030057/s1, File S1: Research protocol; File S2: COREQ and MMAT; File S3: List of codes.

## References

[1] Galbraith K., Ward A., Heneghan C. (2017). A Real-World Approach to Evidence-Based Medicine in General Practice: A Competency Framework Derived from a Systematic Review and Delphi Process. BMC Med. Educ..

[2] Wang T., Tan J.-Y.B., Liu X.-L., Zhao I. (2023). Barriers and Enablers to Implementing Clinical Practice Guidelines in Primary Care: An Overview of Systematic Reviews. BMJ Open.

[3] Otto C.M., Abdullah A.R., Davis L.L., Ferrari V.A., Fremes S., Mukherjee D., Prestera L., Ziaeian B. (2026). 2025 ACC/AHA Clinical Practice Guidelines Core Principles and Development Process: A Report of the American College of Cardiology/American Heart Association Joint Committee on Clinical Practice Guidelines. J. Am. Coll. Cardiol..

[4] Qaseem A., Kansagara D., Lin J.S., Mustafa R.A., Wilt T.J., for the Clinical Guidelines Committee of the American College of Physicians (2019). The Development of Clinical Guidelines and Guidance Statements by the Clinical Guidelines Committee of the American College of Physicians: Update of Methods. Ann. Intern. Med..

[5] Piggott T., Baldeh T., Akl E.A., Junek M., Wiercioch W., Schneider R., Langendam M.W., Meerpohl J., Brozek J.L., Schünemann H.J. (2021). Supporting Effective Participation in Health Guideline Development Groups: The Guideline Participant Tool. J. Clin. Epidemiol..

[6] Mikkola I., Komulainen J., Hagnäs M., Sipilä R. (2025). Importance, Facilitators and Barriers for Finnish General Practitioner Involvement in Clinical Practice Guideline Panels: A Survey. BMC Prim. Care.

[7] Allan G.M., Aubrey-Bassler K., Cauchon M., Ivers N.M., Katz A., Kirkwood J., Kuling P.J., Mang E.J., Moore S., Safarov A. (2021). Endorsement of Clinical Practice Guidelines: Criteria from the College of Family Physicians of Canada. Can. Fam. Physician.

[8] O’Brien E., Duffy S., Harkins V., Smith S.M., O’hErlihy N., Walsh A., Clyne B., Wallace E. (2024). A Scoping Review of Evidence-Based Guidance and Guidelines Published by General Practice Professional Organizations. Fam. Pract..

[9] Forsetlund L., O’Brien M.A., Forsén L., Mwai L., Reinar L.M., Okwen M.P., Horsley T., Rose C.J. (2021). Continuing Education Meetings and Workshops: Effects on Professional Practice and Healthcare Outcomes. Cochrane Database Syst. Rev..

[10] Rohrbasser A., Wong G., Mickan S., Harris J. (2022). Understanding How and Why Quality Circles Improve Standards of Practice, Enhance Professional Development and Increase Psychological Well-Being of General Practitioners: A Realist Synthesis. BMJ Open.

[11] Stacey D., Lewis K.B., Smith M., Carley M., Volk R., Douglas E.E., Pacheco-Brousseau L., Finderup J., Gunderson J., Barry M.J. (2024). Decision Aids for People Facing Health Treatment or Screening Decisions. Cochrane Database Syst. Rev..

[12] Heath I. (2004). View of Health Technology Assessment from the Swampy Lowlands of General Practice. Int. J. Technol. Assess. Health Care.

[13] Abdelhamid A., Howe A., Stokes T., Qureshi N., Steel N. (2014). Primary Care Evidence in Clinical Guidelines: A Mixed Methods Study of Practitioners’ Views. Br. J. Gen. Pract. J. R. Coll. Gen. Pract..

[14] NICE Healthcare Professionals. https://www.nice.org.uk/shaping-our-work.

[15] Carter D., Laka M., Gao Y., Choi O., Tamblyn D., Merlin T. (2025). Engaging Stakeholders along Health Technology Assessment Pathways: A Scoping Review of International Practice. Int. J. Technol. Assess. Health Care.

[16] UEMO. https://uemo.be.

[17] EU Regulation on HTA 2021/2282. https://eur-lex.europa.eu/eli/reg/2021/2282/oj/eng.

[18] UEMO Strengthens Cooperation with EMA, EFPC and WONCA. https://uemo.be/news-uemo/stronger-primary-care-in-the-eu-uemo-strengthens-cooperation-with-ema-efpc-and-wonca-europe/.

[19] WONCA Europe. https://www.woncaeurope.org.

[20] SIG WONCA Cancer and Palliative Care. https://www.globalfamilydoctor.com/groups/SpecialInterestGroups/CancerandPalliativecare.aspx.

[21] EAA. https://www.euaac.org.

[22] Perfors I.A.A., May A.M., Boeijen J.A., De Wit N.J., Van Der Wall E., Helsper C.W. (2019). Involving the General Practitioner during Curative Cancer Treatment: A Systematic Review of Health Care Interventions. BMJ Open.

[23] Abma I.L., Roelofs L.C.G., van der Kolk M.B., Mulder S.F., Schers H.J., Hermens R.P.M.G., van der Wees P.J. (2022). Roles of General Practitioners in Shared Decision-Making for Patients with Cancer: A Qualitative Study. Eur. J. Cancer Care.

[24] Druel V., Gimenez L., Paricaud K., Delord J.-P., Grosclaude P., Boussier N., Bugat M.-E.R. (2020). Improving Communication between the General Practitioner and the Oncologist: A Key Role in Coordinating Care for Patients Suffering from Cancer. BMC Cancer.

[25] Murray S.A., Boyd K., Moine S., Kendall M., Macpherson S., Mitchell G., Amblàs-Novellas J. (2024). Using Illness Trajectories to Inform Person Centred, Advance Care Planning. BMJ.

[26] Meiklejohn J.A., Mimery A., Martin J.H., Bailie R., Garvey G., Walpole E.T., Adams J., Williamson D., Valery P.C. (2016). The Role of the GP in Follow-up Cancer Care: A Systematic Literature Review. J. Cancer Surviv. Res. Pract..

[27] Lang V., Walter S., Fessler J., Koester M.J., Ruetters D., Huebner J. (2017). The Role of the General Practitioner in Cancer Care: A Survey of the Patients’ Perspective. J. Cancer Res. Clin. Oncol..

[28] Pluye P., Quan Nha H. (2014). Combining the Power of Stories and the Power of Numbers: Mixed Methods Research and Mixed Studies Reviews. Annu. Rev. Public Health.

[29] Quan Nha H., Fàbregues S., Bartlett G., Boardman F., Cargo M., Dagenais P., Gagnon M.-P., Griffiths F., Nicolau B., O’cAthain A. (2018). The Mixed Methods Appraisal Tool (MMAT) version 2018 for information professionals and researchers. Educ. Inf..

[30] Tong A., Sainsbury P., Craig J. (2007). Consolidated Criteria for Reporting Qualitative Research (COREQ): A 32-Item Checklist for Interviews and Focus Groups. Int. J. Qual. Health Care.

[31] (2024). WONCA, Dublin. https://www.globalfamilydoctor.com/Conferences/29thWONCAEuropeConference2024.aspx.

[32] UEMO Presentation at WONCA Dublin. https://padlet.com/patrickouvrard1/hta-uemo-wonca-j8mzxli3xfpa1yic.

[33] Pope C. (2009). Qualitative Research in Health Care.

[34] Boers M., Boer A., Harmsen R. (2020). Governance of European Cooperation Processes in Health Technology Assessment: Networking, Paving the Way to Convergence of Practices?.

[35] Smith V., Warty R., Nair A., Krishnan S., Sursas J.A., Costa F.d.S., Vollenhoven B., Wallace E.M. (2019). Defining the Clinician’s Role in Early Health Technology Assessment during Medical Device Innovation—A Systematic Review. BMC Health Serv. Res..

[36] Lips P., Timmers L., Bal R., Delnoij D. (2022). Involvement of Patients and Medical Professionals in the Assessment of Relative Effectiveness: A Need for Closer Cooperation. Value Health.

[37] Van Haesendonck L., Ruof J., Desmet T., Van Dyck W., Simoens S., Huys I., Giuliani R., Toumi M., Dierks C., Dierks J. (2023). The Role of Stakeholder Involvement in the Evolving EU HTA Process: Insights Generated through the European Access Academy’s Multi-Stakeholder Pre-Convention Questionnaire. J. Mark. Access Health Policy.

[38] Rich P. (2018). Low Awareness about CADTH among Practising Doctors in Canada. Can. Med. Assoc. J..

[39] Rosen R., Gabbay J. (1999). Linking Health Technology Assessment to Practice. BMJ (Clin. Res. Ed.).

[40] Petkovic J., Magwood O., Lytvyn L., Khabsa J., Concannon T.W., Welch V., Todhunter-Brown A., Palm M.E., Akl E.A., Mbuagbaw L. (2023). Key Issues for Stakeholder Engagement in the Development of Health and Healthcare Guidelines. Res. Involv. Engagem..

[41] Baradaran A., Tolentino R., Grad R., Ganache I., Gore G., Rahimi S.A., Pluye P. (2024). Outcomes of Guidelines from Health Technology Assessment Organizations in Community-Based Primary Care: A Systematic Mixed Studies Review. Int. J. Technol. Assess. Health Care.

[42] Julian E., Belleman T., Garcia M.J., Mölken M.R.-V., Doeswijk R., Giuliani R., Wörmann B.J., Widmer D., Tilleul P., Arroyo R.C. (2025). Avoiding Error and Finding the Right Balance in European Health Technology Assessments: Insights Generated by the European Access Academy. J. Mark. Access Health Policy.

[43] Ballantine B., Devonald B. (2006). Modern Regulatory Impact Analysis: The Experience of the European Union. Regul. Toxicol. Pharmacol. RTP.

[44] Smith K.E., Fooks G., Collin J., Weishaar H., Gilmore A.B. (2010). Is the Increasing Policy Use of Impact Assessment in Europe Likely to Undermine Efforts to Achieve Healthy Public Policy?. J. Epidemiol. Community Health.

[45] Dufour G., Weeks L., De Angelis G., Marchand D.K., Kaunelis D., Severn M., Walter M., Mittmann N. (2022). How We Might Further Integrate Considerations of Environmental Impact When Assessing the Value of Health Technologies. Int. J. Environ. Res. Public Health.

[46] Pinho-Gomes A.-C., Yoo S.-H., Allen A., Maiden H., Shah K., Toolan M. (2022). Incorporating Environmental and Sustainability Considerations into Health Technology Assessment and Clinical and Public Health Guidelines: A Scoping Review. Int. J. Technol. Assess. Health Care.

[47] Morgan R.L., Kelley L., Guyatt G.H., Johnson A., Lavis J.N. (2018). Decision-Making Frameworks and Considerations for Informing Coverage Decisions for Healthcare Interventions: A Critical Interpretive Synthesis. J. Clin. Epidemiol..

[48] Herrera C.A., Lewin S., Paulsen E., Ciapponi A., Opiyo N., Pantoja T., Rada G., Wiysonge C.S., Bastías G., Marti S.G. (2017). Governance Arrangements for Health Systems in Low-Income Countries: An Overview of Systematic Reviews. Cochrane Database Syst. Rev..

[49] Levy J.I. (2021). Accounting for Health Risk Inequality in Regulatory Impact Analysis: Barriers and Opportunities. Risk Anal..

[50] Booth A., Chowaniec M., Goyal S., Faulkner S., Shaw S. (2025). The Carbon Footprints of Single-Use and Reusable Medical Devices: A Systematic Review. BMJ Open.

[51] Zarro M., Ferranti A., Garagozzo G., Fico G., Pecchia L. (2026). Mapping the Regulatory Landscape for Environmental Sustainability of Medical Device Practices within the European Union. Eur. J. Public Health.

[52] Fraser N., Honneth A., Golb J., Golb J.D. (2003). Redistribution or Recognition? A Political-Philosophical Exchange.

[53] Garcia-Zamora S., Da-Silva F.T., Labudovic I., Vicco M.H. (2026). Bringing Regulatory-Style Benefit-Risk Assessment to the Bedside: A Structured Evidence Synthesis Methodology for Shared Decision-Making. J. Eval. Clin. Pract..

[54] Nicolet J., Mueller Y., Paruta P., Boucher J., Senn N. (2022). What Is the Carbon Footprint of Primary Care Practices? A Retrospective Life-Cycle Analysis in Switzerland. Environ. Health Glob. Access Sci. Source.

[55] Sturmberg J., Topolski S. (2014). For Every Complex Problem, There Is an Answer That Is Clear, Simple and Wrong: And Other Aphorisms about Medical Statistical Fallacies. J. Eval. Clin. Pract..

[56] Zoccali C., Tripepi G. (2026). Real-World Evidence and Observational Studies: Methodological Challenges in Clinical Research. Eur. J. Clin. Investig..

[57] Kympouropoulos S. (2023). Real World Evidence: Methodological Issues and Opportunities from the European Health Data Space. BMC Med. Res. Methodol..

[58] Fricker M. (2007). Epistemic Injustice: Power and the Ethics of Knowing.

[59] Thomas A., Kuper A., Chin-Yee B., Park M. (2020). What Is “Shared” in Shared Decision-making? Philosophical Perspectives, Epistemic Justice, and Implications for Health Professions Education. J. Eval. Clin. Pract..

[60] Hornyak-Bell E., Thomas A., Chrestensen A., Quaiattini A., Lavoie P., Caty M., Drolet M.-J., Rochette A., Kinsella E.A. (2026). Epistemic Injustice in Healthcare Professional Practice: A Scoping Review. Soc. Sci. Med..

[61] De Maeseneer J.M., van Driel M.L., Green L.A., van Weel C. (2003). The Need for Research in Primary Care. Lancet.

[62] Rochoy M. (2026). The European Atlas of General Practice.

[63] Balint M. (2000). The Doctor, His Patient and the Illness.

[64] Gadamer H.-G. (1996). The Enigma of Health: The Art of Healing in a Scientific Age.

[65] Good B.J. (2008). Medicine, Rationality, and Experience: An Anthropological Perspective.

