# Supplementary material for: Role of General Practitioners in European Health Technology Assessment, EU-HTA Innovation in Oncology as an Example
Source: J Mark Access Health Policy. 2026 Sep 21;14(3):57. doi: 10.3390/jmahp14030057 (PMC13608814; doi:10.3390/jmahp14030057)
Supplement: Supplementary file 1 [file jmahp-14-00057-s001.zip › File S2. COREQ and MMAT.pdf]

COREQ (COnsolidated criteria for REporting Qualitative research) Checklist

A checklist of items that should be included in reports of qualitative research. You must report the page number in your manuscript where you consider each of the items listed in this checklist. If you have not included this information, either revise your manuscript accordingly before submitting or note N/A.

Topic	Item No.	Guide Questions/Description	Reported on Page No.
**Domain 1: Research team and reflexivity**		cf. methodology	
Personal characteristics			
Interviewer/facilitator	1	Which author/s conducted the interview or focus group?	DW/HMC
Credentials	2	What were the researcher's credentials? E.g. PhD, MD	cf title
Occupation	3	What was their occupation at the time of the study?	"
Gender	4	Was the researcher male or female? 6 M 2 F	
Experience and training	5	What experience or training did the researcher have?	p4
Relationship with participants			
Relationship established	6	Was a relationship established prior to study commencement?	cf intro
Participant knowledge of the interviewer	7	What did the participants know about the researcher? e.g. personal goals, reasons for doing the research VEMo members	3–4
Interviewer characteristics	8	What characteristics were reported about the inter viewer/facilitator? e.g. Bias, assumptions, reasons and interests in the research topic	4
**Domain 2: Study design**			
Theoretical framework			
Methodological orientation and Theory	9	What methodological orientation was stated to underpin the study? e.g. grounded theory, discourse analysis, ethnography, phenomenology, content analysis thematic analysis	3-4
Participant selection			
Sampling	10	How were participants selected? e.g. purposive, convenience, consecutive, snowball	4
Method of approach	11	How were participants approached? e.g. face-to-face, telephone, mail, email	4
Sample size	12	How many participants were in the study?	Tabl 3
Non-participation	13	How many people refused to participate or dropped out? Reasons?	0
Setting			
Setting of data collection	14	Where was the data collected? e.g. home, clinic, workplace GA	4
Presence of non-participants	15	Was anyone else present besides the participants and researchers?	–
Description of sample	16	What are the important characteristics of the sample? e.g. demographic data, date VEMO members	4
Data collection			
Interview guide	17	Were questions, prompts, guides provided by the authors? Was it pilot tested? questionnaire	4
Repeat interviews	18	Were repeat inter views carried out? If yes, how many? 2 x	4
Audio/visual recording	19	Did the research use audio or visual recording to collect the data?	4
Field notes	20	Were field notes made during and/or after the inter view or focus group?	NO
Duration	21	What was the duration of the inter views or focus group?	90 mn
Data saturation	22	Was data saturation discussed? 4 codes more at 2d FG	4
Transcripts returned	23	Were transcripts returned to participants for comment and/or	4

Topic	Item No.	Guide Questions/Description	Reported on Page No.
		correction?	
**Domain 3: analysis and findings**			
Data analysis			
Number of data coders	24	How many data coders coded the data? 2 coders	R°4
Description of the coding tree	25	Did authors provide a description of the coding tree?	Tabl 2-3
Derivation of themes	26	Were themes identified in advance or derived from the data? no	4
Software	27	What software, if applicable, was used to manage the data? MaxqDA	4
Participant checking	28	Did participants provide feedback on the findings?	4
Reporting			
Quotations presented	29	Were participant quotations presented to illustrate the themes/findings? Was each quotation identified? e.g. participant number	Tabl 2-3
Data and findings consistent	30	Was there consistency between the data presented and the findings?	DISC
Clarity of major themes	31	Were major themes clearly presented in the findings?	Tabl 2-3 +4
Clarity of minor themes	32	Is there a description of diverse cases or discussion of minor themes?	Tab1

Developed from: Tong A, Sainsbury P, Craig J. Consolidated criteria for reporting qualitative research (COREQ): a 32-item checklist for interviews and focus groups. *International Journal for Quality in Health Care*. 2007. Volume 19, Number 6: pp. 349 – 357

Once you have completed this checklist, please save a copy and upload it as part of your submission. DO NOT include this checklist as part of the main manuscript document. It must be uploaded as a separate file.

Part I: Mixed Methods Appraisal Tool (MMAT), version 2018

Category of study designs	Methodological quality criteria	Responses			
		Yes	No	Can't tell	Comments
Screening questions (for all types)	S1. Are there clear research questions? YES	X			title
	S2. Do the collected data allow to address the research questions?	X			conclusion
	Further appraisal may not be feasible or appropriate when the answer is 'No' or 'Can't tell' to one or both screening questions.				
1. Qualitative	1.1. Is the qualitative approach appropriate to answer the research question?	X			
	1.2. Are the qualitative data collection methods adequate to address the research question?	X			COREQ
	1.3. Are the findings adequately derived from the data?	X			
	1.4. Is the interpretation of results sufficiently substantiated by data?	X			quotations
	1.5. Is there coherence between qualitative data sources, collection, analysis and interpretation?	X			results
2. Quantitative randomized controlled trials	2.1. Is randomization appropriately performed? no randomization			–	
	2.2. Are the groups comparable at baseline? no comparison			–	
	2.3. Are there complete outcome data?	X			
	2.4. Are outcome assessors blinded to the intervention provided?		X		*
	2.5 Did the participants adhere to the assigned intervention?	X			
3. Quantitative non-randomized	3.1. Are the participants representative of the target population? UEMO population	X			
	3.2. Are measurements appropriate regarding both the outcome and intervention (or exposure)?	X			
	3.3. Are there complete outcome data?	X			
	3.4. Are the confounders accounted for in the design and analysis?	X			**
	3.5. During the study period, is the intervention administered (or exposure occurred) as intended?				
4. Quantitative descriptive	4.1. Is the sampling strategy relevant to address the research question?	X			
	4.2. Is the sample representative of the target population?	X			
	4.3. Are the measurements appropriate?	X			
	4.4. Is the risk of nonresponse bias low?	X			
	4.5. Is the statistical analysis appropriate to answer the research question?	X			
5. Mixed methods	5.1. Is there an adequate rationale for using a mixed methods design to address the research question?	X			
	5.2. Are the different components of the study effectively integrated to answer the research question?	X			Tabl. 4
	5.3. Are the outputs of the integration of qualitative and quantitative components adequately interpreted?	X			"
	5.4. Are divergences and inconsistencies between quantitative and qualitative results adequately addressed?	X			***
	5.5. Do the different components of the study adhere to the quality criteria of each tradition of the methods involved?	X			COREQ

* preconceptions of researchers are addressed
** the confounders are the non response of questionnaire with one expressing during FG
*** discussed in analysis of questionnaire
