# Supplementary material for: Role of General Practitioners in European Health Technology Assessment, EU-HTA Innovation in Oncology as an Example
Source: J Mark Access Health Policy. 2026 Sep 21;14(3):57. doi: 10.3390/jmahp14030057 (PMC13608814; doi:10.3390/jmahp14030057)
Supplement: Supplementary file 1 [file jmahp-14-00057-s001.zip › File S3. List of codes Document MAXQDA 2018.pdf]

Liste de codes	Mémo	Segments codifiés	Documents
Liste de codes		73	3
arguments/ fears against participation		0	0
complexity of the process		0	0
take care if limiting to PICO		1	1
take too much responsibilities		3	1
facing to a technophilic world		0	0
quaternary prevention can inhibate innovation		1	1
the GP is present at the end of the process in shared decision		1	1
to be taken as alibi		2	1
tokenism		1	1
too much economic and social questions		1	1
risk of conflicts of interest		1	1
problem with confidentiality		1	1
difficulty with patient's associations		1	1
the problem of cost of the process		0	0
Too much resources for one GP or an association of GPs		4	1
who is going to fund it		3	2
EC don't pay the real cost		4	2
what we expect in return if participation		1	1

Liste de codes	Mémo	Segments codifiés	Documents
it is a niche interest		0	0
only retired drs can have time		1	1
basic GP versus niche interest		1	1
Not forget priorities for GPs		0	0
dont fits into my daily practice		1	1
we have to focus on commonalities		2	2
not our role in oncology		1	1
taking resources to most important topics		3	1
priority on communication the very black hole		1	1
argument pro participation		0	0
distinguish stakeholders from experts - degree of engagement		0	0
importance of both		3	1
only know its exists		1	1
green or red flag for research: to be there		1	1
to be consulted as a stakeholder		1	1
give advice for choosing experts		1	1
possibility to introduce dimensions of GP		0	0
be present to inform politicians		2	1
over and undermedicalization		1	1
defend position of patients		2	1

Liste de codes	Mémo	Segments codifiés	Documents
importance of ethics for GPs		1	1
importance of rare diseases for GPs		1	1
we have to focus on every day practice. Ex. obesity		1	1
take care top experts		1	1
defend quality of life in the process		2	1
role in the scope of our practice for oncology		1	1
introduce holistic dimension in guidelines		3	2
give practical advice and questions		2	1
consider extra overload for GPs		1	1
UEMO no but WONCA		0	0
crucial combination UEMO-WONCA		1	1
about memorandum of understanding		1	1
for participation of national colleges or WONCA		2	1
fear not be part of it		1	1
against participation of UEMO		3	2
better application of guideline if participation		2	1
job possible if training on it: GPSI		3	1
