# Supplementary material for: Role of General Practitioners in European Health Technology Assessment, EU-HTA Innovation in Oncology as an Example
Source: J Mark Access Health Policy. 2026 Sep 21;14(3):57. doi: 10.3390/jmahp14030057 (PMC13608814; doi:10.3390/jmahp14030057)
Supplement: Supplementary file 1 [file jmahp-14-00057-s001.zip › jmahp-4402814 file S1.pdf]

Research protocol.

- First discussion June 2024 at the UEMO-General Assembly in Stavanger about the importance to continue or not to participate to EU-HTA, considering the first evaluations beginning in 2025 about oncology.
- Preparation of a questionnaire together with WONCA sig oncology and EAA
- Testing of the questionnaire in WONCA Dublin Sept. 24 during a work-shop
- Presentation of a questionnaire for UEMO GA Brussels autumn 24. With first focus group
- Analysis of questionnaire and FG for presentation to GA Helsinki June 25 followed by a second FG for discussion of results
- No patients involved, no ethical commission
- Confidentiality according usual UEMO process. Discussions anonymized.
